# Regeneration of the Eye

**Published:** 1879-10

**Authors:** 


Regeneration of The Eye.—According to a French journal, some curious facts have come to light about the regeneration of the eye during experiments made by M. Philipeaux. He has aimed to ascertain whether, on completely emptying the eyes of young rabbits and guineapigs, the vitreous humor would be reorganized, and whether even the crystalline lens would be reproduced. He has been careful not to destroy the crystalline capsule, for experience has shown that in order that an organ should be regenerated a portion of it must be left in its place. A month after the mutilation was effected, the eyes which had been emptied were filled afresh, and the crystalline lens was restored. He operated on twenty-four animals, and in each case the mutilated eye revived. This would seem to show that the optic organ has the same capabilities as the bones; the organic process repairs an injury, and reconstructs, more or less completely, that portion which has been lost.—*Boston Journal of Chemistry.*

